# Influence of Ageing and Post-Processing on the Mechanical and Aesthetic Stability of PA12-Based 3D-Printed Components for Medical Devices

**DOI:** 10.3390/ma18194478

**Published:** 2025-09-25

**Authors:** Andrzej Zakręcki, Jacek Cieślik

**Affiliations:** The Faculty of Mechanical Engineering and Robotics, AGH University in Krakow, Al. Mickiewicza 30, 30-059 Kraków, Poland; zakrecki@agh.edu.pl

**Keywords:** additive manufacturing, 3D printing, polyamide PA12, SLS, HP MJF, DyeMansion, ageing test, mechanical properties, color change, medical product

## Abstract

This study investigates the mechanical performance of polyamide 12 (PA12) components fabricated using Selective Laser Sintering (SLS) and HP Multi Jet Fusion (HP MJF) technologies, with particular emphasis on the effects of DyeMansion post-processing techniques. The primary objective was to evaluate the long-term durability of additively manufactured parts intended for use in medical environments, with ageing simulated over a 12-month period. Experimental findings indicate that specimens produced via SLS exhibit superior resistance to physicochemical degradation processes compared with those manufactured using HP MJF. Moreover, industrial dyeing with the DM60 system was found to significantly contribute to the retention of mechanical properties over time. Notably, the SLS-processed PA2200 material demonstrated enhanced mechanical stability after 12 months, particularly in the dyed configuration. These results highlight the critical role of both manufacturing technology and post-processing strategies in ensuring the long-term reliability of PA12 components, especially in applications subject to stringent mechanical and environmental requirements, such as in the medical and industrial sectors.

## 1. Introduction

Three-dimensional printing, also known as additive manufacturing (AM), is a rapidly developing technology that has revolutionized many sectors, including the biomedical industry [1,2,3,4,5,6,7,8]. In medical applications, 3D printing offers several unique advantages, including the ability to produce customized parts quickly and cost-effectively, as well as the integration of different functions and materials into a single structure [9,10,11,12].

In 3D printing, biocompatible products are produced from various polymers, including polymethyl methacrylate (PMMA), thermoplastic polyurethane (TPU) [13] processed using Fused Filament Fabrication (FFF) technology [14], silicone using Liquid 3D Printing technology [15], Bio-Med Clear 3D resins [16] in stereolithography (SLA) [17], and polyamides PA11 and PA12 and TPU [18] in selective laser sintering (SLS) [19] and Multi Jet Fusion (MJF) [20] technologies. Key application areas include implants and prostheses, dentistry (crowns, bridges, diagnostic models, surgical guides, orthodontic trays, and retainers), the production of surgical instruments and orthoses [21,22,23,24,25,26], cranioplasty implants [27], and wearable biosensors [28] and smart wristbands [29,30].

The particular value of 3D printing in medicine is the ability to customize the device to the patient’s anatomy, which significantly affects the effectiveness of therapy, comfort of use, and recovery time. The technology also enables the design and production of complex geometries while maintaining cost-effectiveness for low-volume production and custom devices [31,32,33,34,35,36].

One of the most commonly used materials in medical 3D printing is polyamide 12 (PA12) [37], which is particularly suitable for Selective Laser Sintering (SLS) [38,39,40,41,42] and HP Multi Jet Fusion (MJF) [43,44] technologies. Its popularity is due to its favorable mechanical properties, low density, good dimensional stability, low moisture absorption (for a polyamide), and easy availability [45,46]. The leading manufacturers of 3D printers that use SLS technology include EOS [47], Sinterit [48], Formlabs [49], and 3D Systems [50]. HP is the leader in MJF technology [51]. PA12 is the most commonly used powder in both technologies due to its ease of processing, relatively low price, and high mechanical strength [52]. The PA12 material-designated PA2200, which is used in EOS devices, is certified for biocompatibility according to EN ISO 10993 [53]. In contrast, the PA12 used by HP is certified according to ISO 10993, as well as FDA and USP Class I-VI [54].

A rough and matte surface characterizes parts made of PA12 printed by SLS or MJF, which have a porous structure [43]. Although they already exhibit good mechanical properties in their raw state, many medical applications require additional smoothing and refinement of the surface. Smoothness, uniformity, and surface integrity are critical due to the need to maintain hygiene, minimize the risk of irritation, and reduce bacterial colonization [55]. Post-processing also enhances aesthetics (e.g., achieving uniform color, gloss) and functional properties (e.g., UV and moisture resistance, and tightness). The most commonly used methods include staining, chemical or mechanical smoothing, and coating. Companies such as DyeMansion [56] and AMT [57] specialize in automating these processes, offering industrial solutions with ISO 10993 biocompatibility certification [53] for PA12 materials used in EOS and HP printers.

Polyamide 12, like most polyamide plastics, is susceptible to ageing due to environmental factors. In the context of medical devices, it is essential to understand how prolonged exposure to UV light, elevated temperatures, and humidity affects the mechanical properties and durability of 3D-printed products made from PA12 [58,59]. An additional consideration is the issue of reusing finished medical devices. Although many are designed to be disposable, such as personalized surgical guides, reuse is considered, especially when they are expensive or used by the same patient. In this case, it is essential to determine the impact of repeated use and sterilization on their durability and safety. According to ISO 14971: 2019 [60], the manufacturer is required to analyze risks at each stage of the device’s life cycle, including those associated with reuse, especially if the device was initially designed as a single-use device. In the context of Europe’s ageing population, a significant increase in osteoporotic fractures is anticipated, resulting in a greater demand for customized orthopedic solutions [61,62,63]. Due to its manufacturing flexibility and ability to tailor devices to individual patients, 3D printing can play a crucial role in delivering personalized care. In this context, it is essential to incorporate the principles of sustainability—Reduce, Reuse, Recycle [64]—in the assessment of material sustainability and the potential reuse of medical components. Medical devices printed from PA12 can be exposed to cyclical changes in humidity, temperature, UV radiation, or repeated heating, even if they are not continuously exposed to external conditions. In the case of devices not subjected to high mechanical stress, such as orthoses used outdoors, they can be expected to be maintained for several years [65,66]. Meanwhile, the current state of knowledge focuses mainly on the ageing of PA12 powder used in the 3D printing process, including its repeated use, heating in the working chamber, and deterioration of the properties of subsequent prints [67]. This phenomenon manifests itself as an increase in melt viscosity, a decrease in sintering ability, and a reduction in the mechanical quality of the products. However, the effects of environmental ageing on the properties of finished parts have been insufficiently analyzed, especially considering post-processing such as coloring, mechanical smoothing, or chemical smoothing, including their impact on mechanical properties (e.g., tensile strength). Meanwhile, a complete understanding of the production chain—from design, 3D printing, and post-processing to clinical use—is crucial to ensure the quality and safety of medical devices in accordance with current standards and regulations. The existing literature on polyamide PA12 in SLS and MJF technologies mainly concentrates on powders and process parameters; comparisons between SLS and MJF, e.g., Cai [68] and Sillani [69], primarily address the influence of powder ageing and the properties of freshly printed parts, neglecting the long-term ageing of finished, post-processed products. A few exceptions, such as D’Andrea et al. [70], examine the physical ageing of completed MJF parts after 90 days, noting decreases in yield strength and fatigue life, as well as greater variability in results. It is also understood that powder ageing in SLS diminishes the mechanical properties of finished parts (∼15% reduction in tensile strength, ∼20% in bending strength) due to increased viscosity/molecular weight and poorer sintering [71]. The work of Gruber [72] suggests that additional powder processing (e.g., spheroidization) may alter the functionality of the final component. Simultaneously, there is a lack of data on the long-term effects of mechanical processing combined with coloring and chemical smoothing on the mechanical and color stability (ΔE) of PA12 parts. Aside from reports on annealing [73], no studies explore the actual service life cycles of medical components.

Despite the increasing use of additive manufacturing technologies in medicine, there is still a lack of comprehensive data on the impact of ageing on the efficacy and safety of PA12 components [74]. Another challenge is the development of a consistent process for the design of a personalized medical device, taking into account the diagnosis of the clinical problem, the selection of the 3D printing material and technology, the choice of finishing method, design taking biomechanics into account, validation through strength analysis and digital twin, and finally, market implementation. This publication presents the results of a study on the mechanical properties of PA12 manufactured by the SLS and HP MJF methods, including DyeMansion post-processing techniques. This research aims to determine the durability of components intended for use in a medical environment over up to 12 months. Additionally, an integrated workflow for producing a medical device is presented, utilizing the Industry 4.0 concept. The developed research results can be applied to evaluate the durability of a medical device.

## 2. Materials and Methods

### 2.1. Methods of Making Test Samples

When designing medical components manufactured using 3D printing technology, the choice of material and production technology is crucial. It directly affects structural strength, mechanical properties such as bendability or deformability, and the ability to produce minimum features, from wall thickness to the finest details. Moreover, the selection of material and technology influences how easily the product can be placed on the market, in accordance with the requirements of the Medical Device Regulation (MDR) and the European Union’s medical device approval procedures [75]. Consequently, all these factors have a direct impact on the final geometry and weight of the designed component.

In this study, the commonly used PA12 polyamide, used in SLS and HP MJF technologies, was used, using two types of polyamide powder: PA2200 (EOS’s trade name for white PA12) [76] for SLS and PA12 [77] for HP MJF, both certified for biocompatibility according to ISO 10993. PA2200 is a white 12-based polyamide powder developed by EOS specifically for use in selective laser sintering technology [78,79,80]. PA12 HP 3D High Reusability is a tough thermoplastic specifically designed for Multi Jet Fusion technology [81]. Information on the material properties was obtained from the respective material data sheets. The characteristics of the polyamide powders used are presented in Table 1. Data on the properties of PA2200 and PA12 HP 3D High Reusability are shown in Table 2. For SLS technology, samples were prepared in accordance with ISO standards, covering tensile, bending, and impact test specimens, and then tested for mechanical and thermal properties and biocompatibility. For HP MJF technology, samples were produced using the manufacturer’s material profile and tested according to ASTM standards, analyzing mechanical strength, elasticity modules, impact resistance, thermal properties, and compliance with FDA and USP biocompatibility requirements.

The PA22000 samples were produced on an EOS P396 [83], while the PA12 HP 3D High Reusability samples were made on an HP 5200 [84]. EOS P396 requires manual powder loading and a long cooling time in the chamber (10–20 h). The total cycle time is approximately 34 h [52]. The HP Jet Fusion 5200 features greater process automation and does not require process gases, and offers automatic powder mixing and an external cooling unit (6–8 h). The system is equipped with an automatic unpacking station, which significantly reduces post-processing time. The total cycle time is approximately 16 h [52]. Each of the 3D printers–EOS P396 and HP 5200–has a default set of process parameters assigned to a specific material profile. For EOS P396, the PA2200 material profile was used, selected in the EOSPRINT 2.13 software from a built-in library of predefined profiles. For HP 5200, the HP 3D HR PA12 material profile was used in the HP SmartStream 3D v2.0 software.The material profile is selected automatically through an intelligent material cartridge recognition system, followed by the application of pre-certified profiles available in the HP ecosystem. The process parameters of the equipment used are shown in Table 3.

The samples, which were made from PA12 polyamide using SLS and HP MJF technologies, were then finished with the DyeMansion system using the following equipment:

- Powershot C for cleaning parts from unbaked powder (PolyShot Cleaning). The Powershot C is the industry standard for automatic cleaning of parts from 3D printers using SLS, HP MJF, and HSS technologies [85].
- Powershot S for surface finishing (PolyShot Surfacing). Powershot S is an advanced surface finishing system that uses PolyShot Surfacing (PSS) technology [86].
- DM60 for industrial coloring (DeepDye Coloring). DM60 is the leading industrial coloring solution for 3D printing, offering an unlimited range of color possibilities [87].
- Powerfuse S for vapor polishing (VaporFuse Surfacing). Powerfuse S is an environmentally friendly vapor polishing system designed for industrial surface finishing [88].

For the dyeing process, it was decided to use the standard black color designated Black 01 for both SLS and HP MJF parts. The operating parameters of the DyeMansion’s devices, along with their default settings for processing components made of polyamide PA12, are shown in Table 4.

### 2.2. Preparation and Manufacture of Specimens

**Figure 1:**
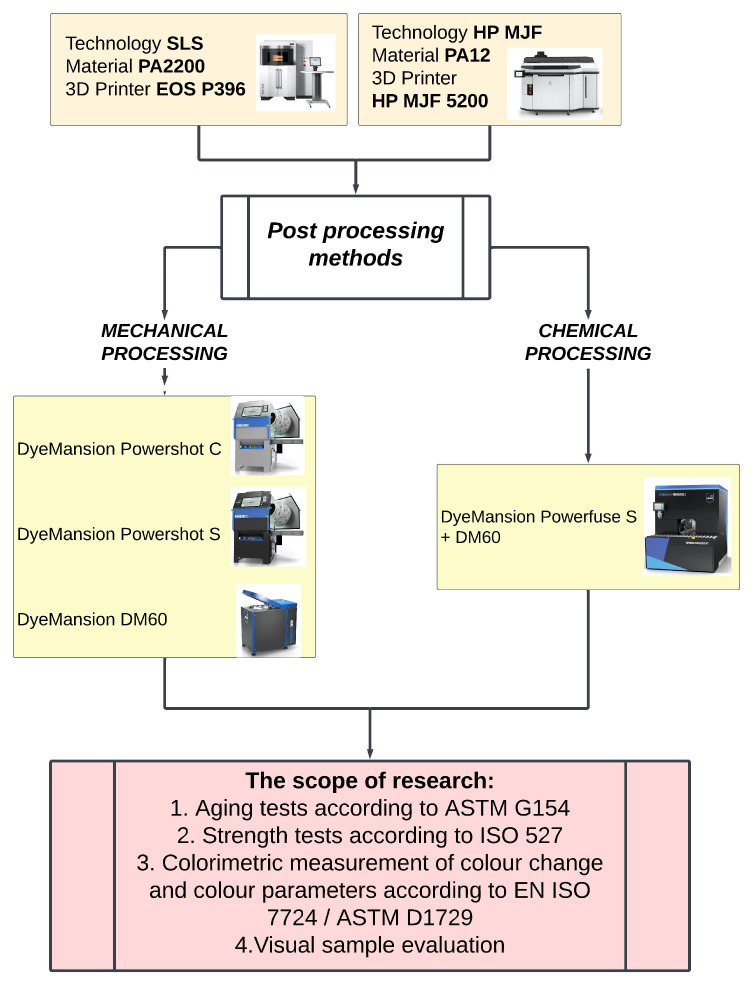
Schematic of the testing of PA12 polymer in the context of its use in production as a medical device.

To evaluate the mechanical properties of the PA12 polyamide specimens, tensile specimens were prepared in accordance with ISO 527 [89]. The specimens were printed in horizontal orientation in three different configurations, depending on the 3D printing technology used and the post-processing treatment. The set of prepared samples was subjected to ageing tests, corresponding to periods of use of 6 and 12 months. Therefore, for each combination of printing technology, post-processing route, and ageing time, we tested 6 tensile strength specimens. The choice of 6 (above the ISO 527 minimum of 5) was made to increase statistical power, given the known inter-specimen variability in AM parts, and to prevent data loss if a single specimen had to be rejected. The tensile test specimens measured 160 mm (length) × 20 mm (width) × 4 mm (height). In addition, colorimetric color measurement and a tensile test were carried out. A schematic of the tests carried out is shown in Figure 1.

**Figure 2:**
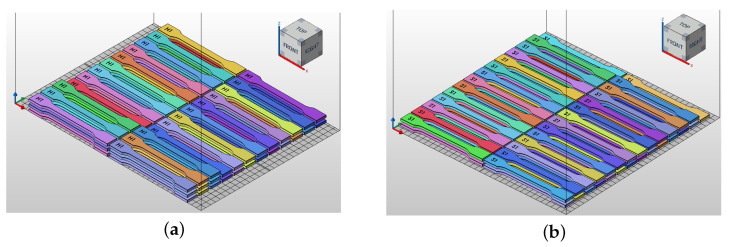
The impact of sample orientation on HP MJF and SLS additive manufacturing within the working chamber, demonstrated in the Autodesk Netfabb environment. Preparation of samples for production from PA12 polyamide material on HP MJF 5200 (**a**) and EOS P396 machines (**b**). Arrangement of samples for tensile strength testing in accordance with ISO 527 inside the machine’s working chamber.

The specimens were produced using EOS P396 and HP 5200. The samples were produced using the PA2200 material profile assigned to the EOS P396 device and the PA12 HP 3D High Reusability material profile assigned to HP 5200. These are the standard process parameters for manufacturing PA12 polyamide components. The samples in the EOS device were positioned in the XY plane (X being the longest side of the element) on the platform, covering an area of 335 mm along the X axis and 322 mm along the Y axis, with a Z height of 22 mm. The samples in the HP device were placed in the XY plane on the platform, covering an area of 352 mm along the X axis and 284 mm along the Y axis, with a height of 22 mm. In the EOS P396 device, the base of the working chamber is square, whereas in HP 5200, it is rectangular. Hence, it is crucial to arrange the elements within the chamber so that the longest edge of the printed 3D element aligns with the chamber’s longest edge. The arrangement of the specimens in the working chamber of both devices is shown in Figure 2.

**Figure 3:**
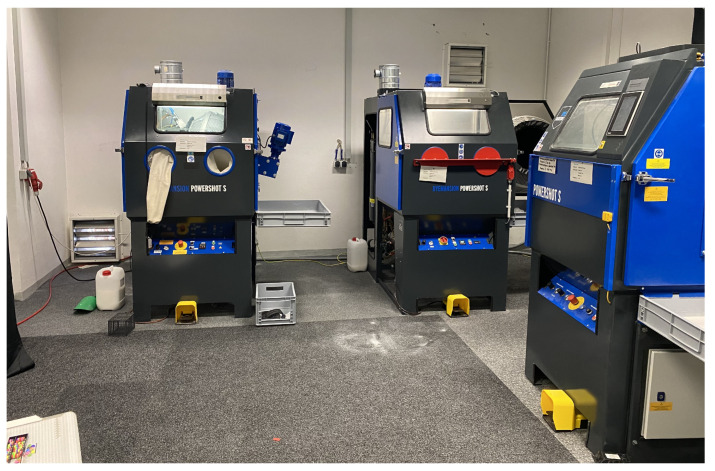
DyeMansion post-processing system presenting three Powershot S machines.

The specimens were then subjected to post-processing using DyeMansion equipment, as specified in the configuration shown in Table 5. DyeMansion’s devices are shown in Figure 3.

### 2.3. Ageing Tests and Methods for Assessing Sample Properties

Ageing tests [90,91,92] were carried out in a Q-Lab Q-SUN Xenon Test Xe-3 chamber [93], equipped with xenon lamps, according to the following cyclic program:

- Air temperature: 38 °C;
- Black standard temperature (IBP): 55 °C;
- Mixed cycle: dry irradiation/irradiation with relative humidity of 50% RH;
- UV irradiance: 60 W/m^2^ (TUV spectral range 300–400 nm).

The samples were placed in a climate chamber, evenly spread across the entire working surface to ensure consistent exposure conditions in accordance with the equipment manufacturer’s guidelines and ISO 4892 [90]. The study was divided into the following two stages (test time in the chamber):

1. Stage 1: 375 h—corresponding to approximately 6 months of ageing under natural conditions;
2. Stage 2: 750 h—corresponding to approximately 12 months of ageing under natural conditions.

To calculate the aging time in a climate chamber corresponding to the actual time equivalent (6 and 12 months), we used the Accelerated Aging Calculator, entering the following data: Temperature of Accelerated Aging Environment—55 °C, Ambient Room Temperature—23 °C, Aging Factor—2.15. Based on these values, the Calculated Accelerated Aging Time was determined: for 6 months, it was approximately 15.76 days (∼375 h), and for 12 months, it was approximately 31.5 days (∼750 h).

**Figure 4:**
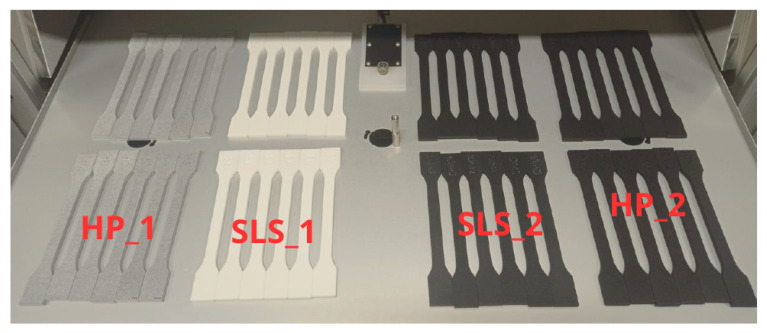
Samples produced for ageing tests and tensile tests; the gray samples are made of PA12 using HP MJF technology with Powershot C post-processing (HP_1); the white samples are made using SLS technology with Powershot C post-processing (SLS_1); the black samples are made using SLS and HP MJF technologies with Powershot C + DM60 + Powershot S post-processing techniques (SLS_2 and HP_2).

The manufactured samples processed with different post-processing methods are shown in Figure 4.

During the tests, a colorimetric color measurement was carried out in accordance with ISO 7724 [94], using the NR60CP colorimeter. The measurement was carried out using D65 illumination and a SCI Ø4 measuring geometry in the CIE L*a*b* color system. Documentation was completed with photographs of reference samples (before ageing) and samples after 375 h and 750 h of exposure. Documentation was completed with photographs of reference samples (before ageing) and samples after 375 h and 750 h of exposure. For each configuration, a tensile test was conducted in accordance with EN ISO 527-1 [89], using an Instron 5969 testing machine with a maximum load capacity of up to 50 kN. The tests were carried out at a speed of 1 mm/min and a test section length of L = 115 mm. The testing machine has a calibration certificate for testing machines issued by the Regional Measurement Office in Poznań, No. OUM4.WM.473.908-1.2003, dated 26 July 2023.

### 2.4. Statistical Analysis Methods

To evaluate the effect of 3D printing technology, post-processing variant techniques, and ageing time on the mechanical properties of PA12, a statistical analysis was performed using three-way ANOVA (3 × 3 × 2) [95], MANOVA [96], PCA [97], post hoc tests, and effect size measures [98]. The main analysis was a three-way ANOVA considering the following:

- Technology: SLS, HP MJF;
- Post-processing methods: Powershot C + S, Powershot C + S + DM60, Powershot C + S + DM60 + Powerfuse S;
- Ageing time (0, 6, 12 months), including interactions.

Assumptions of normality and homogeneity were tested with Shapiro–Wilk and Levene’s tests. As σ, *E*, and ε were correlated, MANOVA was applied to assess effects jointly and reduce Type I error, using Pillai’s Trace and Wilks’s Lambda. PCA reduced dimensionality and visualized degradation trajectories, with PC1 linked to strength/elongation and PC2 to stiffness. Pairwise comparisons used Tukey HSD (equal variances) [99] or Games-Howell (unequal) [100], supported by effect sizes (Cohen’s d, $\eta_{\mathrm{partial}}^{2}$).

## 3. Results and Discussion

### 3.1. Ageing and Tensile Test Results and Discussion

**Figure 5:**
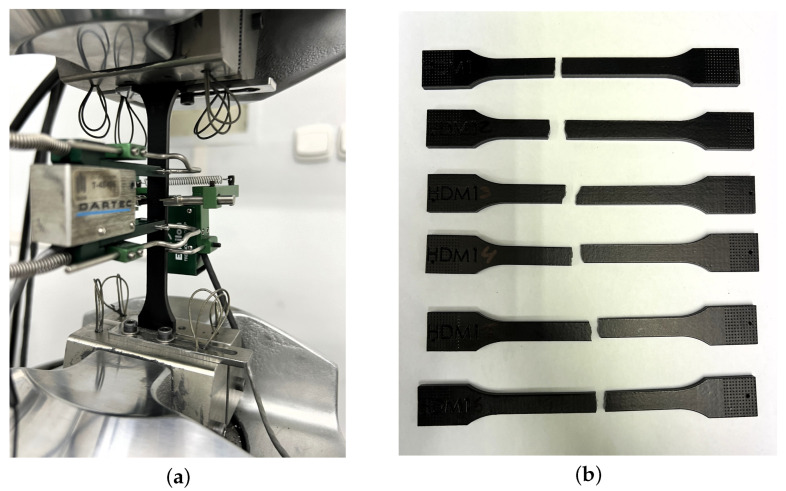
Tensile test of a PA12 sample produced using HP MJF technology with mechanical processing, dyeing, and chemical smoothing in DyeMansion devices (**a**); samples after tensile testing (**b**).

In this study, the effect of the ageing process on the mechanical properties of polyamide PA12 printed using SLS (EOS PA2200) and MJF (HP High Reusability PA12) technologies was assessed. Figure 5 shows the tensile test performed and the specimens from the Powerfuse S chemical post-processing. The specimens were tested at three time points: 0, 6, and 12 months of service. Three surface post-processing options were considered: (1) Powershot C + Powershot S, (2) Powershot C + DM60 + Powershot S, and (3) Powershot C + DM60 + Powershot S + Powerfuse S. Tensile strength (σ), Young’s modulus (E), and elongation at break (ε) were analyzed. The results of the tensile strength parameters for the aged specimens from the tensile tests are shown in Table 6, Table 7 and Table 8.

The biggest differences between the technologies were noted in terms of the loss of mechanical properties due to the ageing process. In the SLS technology for PA2200, the tensile strength remained stable over time for the dyed variant (DM60), with a slight increase in tensile strength to 45 MPa after 12 months. In contrast, for the MJF samples with HP High Reusability PA12, a substantial decrease in tensile strength was observed, as much as 54% for the variant with chemical smoothing in Powerfuse S. These trends are consistent with the results of Puttonen [101], who reported a ∼40% decrease in strength after accelerated ageing of PA12 under UV conditions.

Studies by Machotova [102], Wahab [103], and Weinmann [71] indicate that PA12 parts manufactured using SLS technology exhibit a tensile strength of 43–46 MPa from virgin powder without post-processing. However, ageing and repeated recycling of the powder lead to significant decreases–even below 10 MPa after repeated use. In the case of MJF technology, the initial values are comparable or higher, at approximately 49 MPa (without finishing). More recent studies show that, here as well, powder ageing and physical ageing processes can reduce strength to around 30 MPa, emphasizing the importance of material quality control and the material’s life cycle.

**Figure 6:**
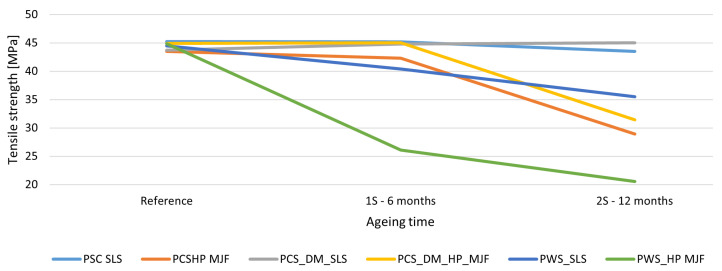
The effect of ageing on tensile strength. Tensile strength of PA12 samples produced by SLS and HP MJF with various post-processing methods after accelerated ageing for 0, 6, and 12 months. SLS samples retained higher tensile strength compared with MJF, which experienced a more significant decline, especially after chemical smoothing. Dyeing slightly stabilized or enhanced the tensile strength of SLS specimens, whereas chemical smoothing accelerated degradation, notably in MJF.

For SLS technology, the loss of strength was minimal across all variants. In the case of the DM60 + S variant, an increase in strength was even noted from 44.0 to 45.0 MPa. The largest reduction among SLS samples was observed in the Powershot C + DM60 + S + Powerfuse S variant from 44.5 to 35.5 MPa, although it remained lower than that in MJF technology. MJF samples exhibited a significant decrease in strength after just 6 months, especially following chemical smoothing from 44.9 to 20.5 MPa after 12 months. The Powershot C+S variant also experienced a notable decline from 44.9 to 28.9 MPa. Components produced using SLS technology demonstrate higher mechanical stability over time, whereas MJF appears highly susceptible to degradation, particularly after chemical smoothing. The effect of the ageing process on tensile strength is shown in Figure 6.

**Figure 7:**
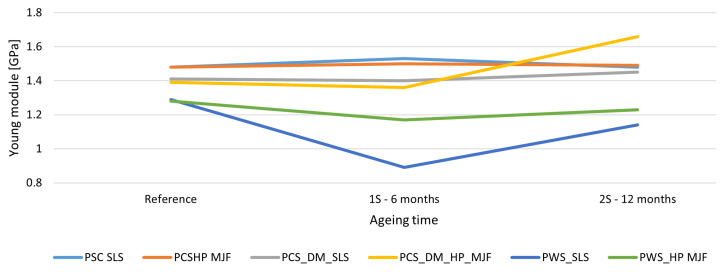
The effect of ageing on Young’s modulus. Young’s modulus of PA12 specimens produced by SLS and HP MJF with different post-processing variants after accelerated ageing for 0, 6, and 12 months. SLS samples after chemical smoothing exhibited a noticeable reduction in stiffness at 6 months, with partial recovery observed after 12 months. In contrast, dyed MJF specimens achieved the highest modulus values after ageing. Overall, SLS maintained more stable stiffness, whereas MJF exhibited a greater increase in modulus over time, correlating with reduced ductility.

Young’s modulus (*E*) remained relatively stable. Changes did not exceed 20% and were in line with the observations of Ghimouz [46], who showed little dependence of Young’s modulus on ageing conditions. The increase in stiffness in some samples (e.g., MJF+DM60) may be a result of the material’s co-crystallization, as also noted in the work of Gazzerro [104] in the context of regranulated PA12 powder. Regarding SLS technology (without post-processing), Young’s modulus of PA12 parts reaches 1600–2000 MPa, but with ageing and powder recycling, a notable decrease occurs, limiting long-term mechanical stability. For MJF technology (without post-processing), Young’s modulus values are comparable or higher (around 1700 MPa), but studies indicate that physical ageing and secondary crystallization can lower the modulus to approximately 1450 MPa, emphasizing the importance of controlling processing and usage. The effect of the ageing process on Young’s modulus is shown in Figure 7.

**Figure 8:**
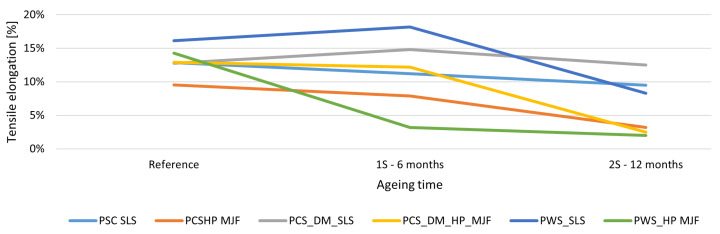
The effect of ageing on tensile elongation. Tensile elongation of PA12 specimens produced by SLS and HP MJF with different post-processing methods was examined after accelerated ageing for 0, 6, and 12 months. All specimens showed a decline in ductility over time, with the most significant decrease observed in chemically smoothed MJF samples. SLS specimens maintained higher elongation values, while MJF specimens experienced a notable loss of plasticity, confirming their increased susceptibility to ageing.

The elongation at break showed the most incredible sensitivity to ageing. MJF samples only achieved ∼2% elongation after 12 months, implying a loss of up to 85% ductility. SLS samples, on the other hand, retained high ductility—especially the colored variants, for which the maximum elongation value remained at ∼12.5%. Puttonen [101] and Ghimouz [46] confirmed that elongation was the most severely degraded parameter due to environmental ageing. The surface finishes used had a significant effect on mechanical durability. DM60 staining had a stabilizing effect, particularly with SLS technology, probably forming a barrier against UV and oxygen. In contrast, chemical smoothing improved initial aesthetics and ductility, but led to faster degradation—especially in MJF technology. Rosso [105], Fiorillo [106], and Zakręcki [52] showed that MJF is more sensitive to property degradation, which explains the observations of a significant decrease in maximum elongation at break and maximum tensile strength. Furthermore, PA12 parts produced using SLS technology (without post-processing) exhibit relatively low elongation at break (5–9%), which decreases further due to powder ageing, although an unusual increase in this parameter has been observed under certain thermal conditions. In MJF technology (without post-processing), the elongation at break is considerably higher (15–20%) and may even increase during physical ageing (to over 30%), indicating better interlayer bonding and greater flexibility of the material compared with SLS. The effect of the ageing process on elongation is shown in Figure 8.

In summary, PA12 printed with SLS technology demonstrates greater resistance to ageing, and the surface finish, achieved through industrial coloring, helps maintain mechanical properties. The MJF technology, although beneficial for production, necessitates additional protective measures for long-term use.

### 3.2. Color Measurement Results

**Figure 9:**
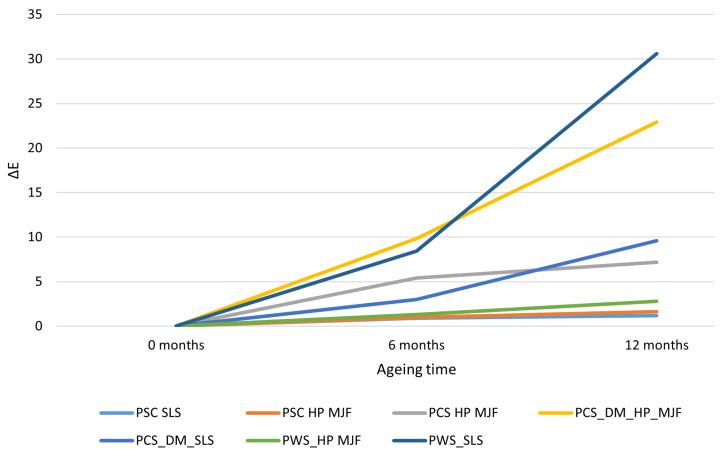
Color change ΔE in subsequent ageing steps. Color change of PA12 specimens manufactured using SLS and HP MJF under different post-processing conditions after accelerated ageing for 0, 6, and 12 months. The most notable discoloration was seen in SLS samples, especially after dyeing, whereas MJF specimens showed significantly lower ΔE values. These findings suggest greater color stability in MJF parts compared with SLS and emphasize the limited protective effect of industrial dyeing and chemical smoothing against long-term ageing.

In the experiments carried out, the color changes of the samples were measured in the CIE L*a*b* system as a difference in color change, ΔE, from the baseline (0 h). The resulting ΔE values for the individual samples (after 375 h and 750 h of exposure) are shown in Figure 9. Minimal color changes (<ΔE=2) were observed in samples subjected solely to cleaning post-processing (Powershot C). For both the white SLS print and the grey HP MJF print, there was a slight color change (ΔE≈1–1.6) after 750 h of exposure. This indicates that the base material without additional dyeing maintains good color stability over the studied period. Such small ΔE color changes suggest that the differences are nearly invisible to the eye. Therefore, the cleaning of the Powershot C did not introduce any factors that accelerate color degradation, and the materials themselves showed no significant tendency to discolor over approximately 750 h (roughly 12 months of simulated exposure).

The graph shown in Figure 9 illustrates the impact of ageing over a 6-month and 12-month reference period, depending on the production method based on 3D printing technology and the finishing method employed, in accordance with the variants presented in Table 5.

**Figure 10:**
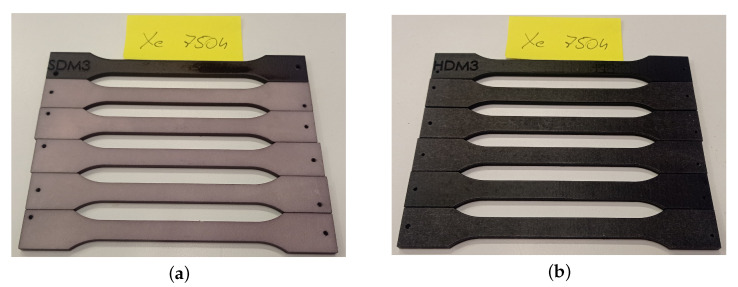
Comparison of color change after 12 months of ageing for samples made using (**a**) SLS technology with Powershot C + S + DM60 finishing and (**b**) HP MJF technology with Powershot C + S + DM60 finishing.

In contrast, notable color changes occurred in samples stained with DyeMansion DM60 following mechanical processing in Powershot S. After just 375 h, significant differences were apparent—particularly for the HP-derived MJF sample (ΔE≈9.8)—while the SLS sample showed a ΔE of approximately 3. By 750 h, both materials had notably faded as the dye degraded under UV light. The MJF sample experienced a greater color change (ΔE≈22.9) compared with the SLS sample (ΔE≈9.6), indicating that initial color intensity and material composition influence the rate of fading. The initially darker or colored MJF component likely lost more color, while the SLS sample benefited from slightly better dye penetration, which slowed the overall discoloration. Despite the manufacturer’s claims of high UV resistance for the DeepDye dyes, results demonstrate that, after long-term exposure (12 months), a noticeable loss of color still occurs, as shown in Figure 10.

The most significant changes were observed in samples subjected to chemical smoothing using the Powerfuse S method, especially those derived from SLS technology. The SLS sample reached ΔE>30 after 750 ageing hours, indicating significant darkening of the entire surface. The chemical smoothing process may have further influenced the progression of this phenomenon. The black MJF material is less susceptible to noticeable discoloration—probably owing to the pigment content acting as a UV stabilizer and masking any yellowing with the dark base color. In other words, the dark shade of the HP MJF material “hides” minor color changes, while the white SLS clearly reveals even slight discoloration.

The effect of exposure time is clearly observable in all cases, as indicated by the increases in ΔE between 375 h and 750 h, which suggest ongoing ageing processes. For samples that initially showed only minor changes, further exposure did not produce a significant visual difference. Conversely, samples with moderate changes after 375 h experienced rapid color deterioration up to 750 h. For instance, the dyed MJF more than doubled ΔE (from approximately 10 to 23), and the chemically smoothed SLS increased about 3.5 times (from roughly 8.4 to 30.6). This illustrates that color degradation can accelerate—initially, the material loses some color, but over time, photochemical mechanisms such as further dye decomposition or polymer oxidation lead to progressively greater changes. Practically, this means that assessing color fastness after a shorter period (e.g., 6 months of ageing) may not fully reveal the more severe deteriorations observed after longer exposure.

### 3.3. Three-Factor ANOVA Analysis

A three-factor ANOVA confirmed the significant influence of all factors considered—3D printing technology, post-processing variant, and ageing time—on the mechanical properties of PA12. The F-statistic values were very high, with corresponding significance levels of p<0.001 for the main effects of each variable (technology, post-processing, ageing time) and most interactions. Partial $\eta^{2}$ indicated a strong practical effect of printing technology, type of post-processing, and ageing time on tensile strength. Similarly, for Young’s modulus (*E*), the post-processing factor was the most significant contributor to explaining the variation, while for elongation at break, printing technology and ageing time dominated. The assumptions of ANOVA were met—the distributions within the groups did not deviate significantly from normality, and the variances could be considered homogeneous, which legitimizes the use of a linear ANOVA model. Table 9 and Table 10 show the result of the statistical ANOVA analysis.

Interactions between factors were also found to be significant, indicating that the effect of one factor depended on the level of another. From an engineering research perspective, the most important relationships are the following:

1. Technology × Ageing process—properties deteriorated significantly more over time for HP MJF samples than for SLS.
2. Technology × Post-processing method—the effectiveness of individual treatments in maintaining mechanical parameters differed between SLS and MJF.
3. Post-processing × Ageing process—the rate of property degradation depended on the type of surface finish.

The three-factor interaction was also statistically significant (p<0.001), indicating the complex, synergistic nature of the influence of the printing process, treatment, and ageing. This makes it possible to answer the following key research questions:

1. Does complete chemical smoothing (Powerfuse S) slow down the degradation of MJF samples more than SLS?
  The results indicate that this is not the case; on the contrary, after 6 and 12 months, MJF samples subjected to smoothing showed a greater decrease in strength and ductility than the corresponding SLS samples (technology × time interaction for the Powerfuse S variant).
2. Does chemical smoothing reduce the mechanical properties of MJF more than SLS?
  Yes, the adverse effect of smoothing (reduction in strength and deformation) was stronger for MJF, especially over a long ageing period.
3. Does mechanical treatment alone (Powershot C + S) protect MJF more effectively than SLS?
  No, after 12 months, the mechanically finished MJF samples lost a significant amount of strength (from 43.5 to 28.9 MPa on average, a decrease of ∼34%), while the corresponding SLS samples remained almost at the same level (45.2→43.5 MPa, a decrease of only ∼4%).
4. Does the addition of dyeing (DM60) improve the ageing resistance of MJF more than SLS?
  No, although staining slightly reduced the degradation rate of MJF (e.g., MJF with staining dropped to 31.4 MPa instead of 28.9 MPa as for unstained samples), SLS samples with dyeing practically did not lose strength during the study period (43.7→45.0 MPa, no decrease).

### 3.4. Multivariate Analysis of Variance

The MANOVA analysis, applied to the correlated response variables (σ, *E*, ε), confirmed significant changes in mechanical property profiles under all studied factors. Considering covariances between strength, stiffness, and elongation, the effects of printing technology, post-processing, and ageing time remained significant. The ageing factor was highly significant, confirming overall degradation with time. Interactions involving technology, treatment, and ageing were also significant in multivariate terms, while the technology × post-processing interaction showed a near-significant trend. The use of MANOVA reduced the risk of Type I error associated with multiple testing of separate ANOVAs and confirmed that the observed differences between SLS and MJF systems as a function of treatment and time relate to the overall mechanical property profile and not just to individual parameters, as shown in Table 11.

### 3.5. Principal Component Analysis

To better understand the patterns of change of the three correlated characteristics, a principal component analysis was conducted. The first two principal components, together, explain approximately 95% of the total variance in the data, enabling the mapping of sample states in 2D space with high accuracy.

As expected, PC1 mainly combines strength and ductility, with high positive loadings for tensile strength and strain and a much smaller contribution from Young’s modulus (*E*). This indicates that PC1 reflects the overall mechanical condition of the material in terms of strength and ductility—specimens with high tensile strength also tended to have high elongation (making them more ’tough’ than brittle), whereas a notable decrease in ductility accompanied a reduction in strength. PC2, on the other hand, primarily correlates with Young’s modulus (*E*) under high loading conditions, with little influence from other variables—it can be seen as a measure of stiffness. Importantly, these components were nearly orthogonal, confirming that, by and large, changes in stiffness occurred independently of simultaneous changes in strength and elongation (for example, specimens could lose ductility and strength without a proportional change in Young’s modulus), as demonstrated in Table 12.

**Figure 11:**
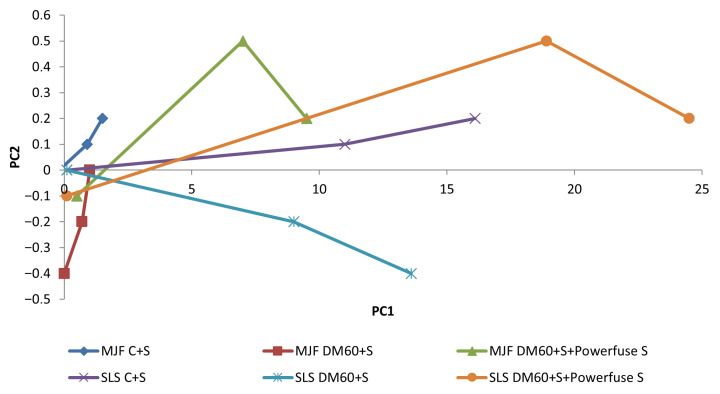
Trajectories of changes in the mechanical properties of PA12 samples in the space of the first two principal components: PC1—represents overall strength and ductility, and PC2—corresponds to stiffness (correlates with Young’s modulus E).

The coordinates of each group of specimens (for each combination of technology and post-processing, at baseline and after 6 and 12 months) were plotted on a graph in the system. PC1 corresponds to strength and ductility, while PC2 corresponds to stiffness and Young’s modulus (*E*), resulting in a map of the property degradation trajectories shown in Figure 11.

Key observations are that SLS samples have short trajectories and minimally degrade, while MJF samples with long trajectories exhibit strong degradation, especially after chemical smoothing. PCA confirms the greater stability of SLS samples, which retain their original properties even after 12 months. MJF trajectories are a clear indicator of degradation—the material loses plastic and strength properties and, in some cases, becomes brittle and stiff.

### 3.6. Post Hoc Tests

Once the significance of the factors’ effect (ANOVA) was established, post hoc procedures were performed to identify significant differences between pairs of groups. The Tukey HSD test, which controls for type I error accumulation, was used. The results revealed all pairs of samples that were statistically different in terms of tensile strength, Young’s modulus, or strain. For example, already after 6 months, the differences in elongation at break between the MJF and SLS samples were significant across all variants (p<0.01), and after 12 months, the strength of the MJF samples also fell significantly below the values recorded for the corresponding SLS samples (p<0.001 for each pair of SLS vs. MJF with the same post-processing variant). Importantly, alongside reporting *p*-values, measures of effect size were also presented—partial $\eta^{2}$ for overall effects and Cohen’s *d* for significant differences between groups. The inclusion of effect sizes enables the practical significance of the results to be assessed. Substantial effect sizes were observed in all key comparisons. For example, the difference in strength between chemically smoothed MJF and SLS samples after 12 months is d≈3.4, while the difference in elongation at break exceeds d=5 (indicating an extremely large property divergence). Additionally, the effect of ageing itself was quantified: for the MJF samples (black variant), the decrease in ductility after 1 year reached d≈4 relative to the initial condition, whereas for the SLS samples, d≈1 (moderate effect). Table 13 displays the results of the post hoc tests conducted.

## 4. Conclusions

To the authors’ knowledge, this is the first systematic experimental study directly comparing SLS and MJF PA12 components for medical devices under controlled long-term ageing conditions.

The aim of this study was to evaluate the impact of 3D printing technologies (SLS and HP MJF), post-processing methods (mechanical cleaning and smoothing—Powershot C/S, dyeing—DM60, chemical smoothing—Powerfuse S), and ageing time (0, 6, and 12 months of exposure in a climate chamber) on the mechanical and aesthetic properties of PA12 components used in medical devices. The results showed that SLS (PA2200 prints) has significantly better resistance to physical degradation over time than HP MJF (PA12 HP 3D High Reusability), particularly in terms of tensile strength and elongation at break. After 12 months, the mechanical properties of SLS remained at a level similar to the initial values (≈44MPa, 8–13%), while HP MJF exhibited significant deterioration (reduction in strength and increased brittleness of the material).

In terms of aesthetics, it was found that exposure time and post-processing significantly influence color stability. The greatest color fastness was observed in uncolored samples, especially dark MJF parts, which retained their color almost unchanged even after 750 h of exposure. Conversely, dyed and white samples produced via SLS showed noticeable color changes, indicating the need to use UV stabilizers or choose darker colors when designing medical devices.

Based on this, the following conclusions were drawn:

- The manufacturing technology is vital for the long-term durability of PA12 components—SLS-printed parts retained most of their mechanical properties after 12 months of exposure, whereas MJF parts exhibited significant degradation and loss of plasticity.
- Post-processing clearly affected the properties—Powershot C+DM60+Powershot S post-processing variant in SLS stabilized strength after ageing, while chemical smoothing (Powerfuse S) caused a short-term increase in ductility at the expense of long-term stability, especially in MJF technology.
- Color stability depended on the technology and post-processing method—dark, uncolored MJF parts demonstrated the highest color fastness, while light and colored SLS parts were more vulnerable to discoloration.
- The results highlight the need for UV stabilizers or darker shades in designs requiring aesthetic durability, especially in medical applications.
- The proposed medical product life cycle (up to 12 months of use with a review after 6 months) ensures safety and functionality while reflecting the actual ageing resistance of the materials.
- The research presented offers valuable data to support the design and selection of technologies for 3D-printed medical devices, combining mechanical and aesthetic analysis in relation to ageing processes.

The results offer practical guidance for medical device designers, emphasizing the importance of carefully choosing manufacturing and finishing technologies within the context of expected mechanical and aesthetic durability. The suggested product life cycle aligns with the test findings and ensures safety and reliability in clinical applications. Direct applications include orthoses, prostheses, and medical equipment components where it is vital to preserve functionality and aesthetics during use. Future research will explore the roughness, hardness, and wear of PA12 components and extend to other biocompatible materials such as PA11 and TPU to assess their long-term suitability for medical devices.

## Figures and Tables

**Table 1:** Parameters of the powders used to produce the samples [79,81].

Parameter	PA2200	PA 12
Average grain-particle size (µm)	56	60
Bulk density (g/cm^3^)	0.45	0.425
Powder melting point (°C)	176	187
Density of parts (g/cm^3^)	0.93	1.01
Powder refresh rate (%)	50	20

**Table 2:** Polyamide PA12 properties for SLS and HP MJF technologies [78,80,81,82].

Properties	PA2200 (SLS)	PA12 (MJF)
Tensile Strength—X/Y Direction (MPa)	48	48
Tensile Strength—Z Direction (MPa)	42	50
Tensile Modulus—X/Y Direction (MPa)	1650	1700–1900
Tensile Modulus—Z Direction (MPa)	1650	1850
Elongation at Break—X/Y Direction (%)	18	17–20
Elongation at Break—Z Direction (%)	4	9–15
Flexural Modulus (MPa)	1500	1700–1800
Charpy Impact Strength (+23 °C) (kJ/m^2^)	53	4.8
Shore D Hardness (–)	75	
Biocompatibility (standards)	EN ISO 10993-1, USP/level VI/121 °C	USP Class I–VI, FDA guidance

**Table 3:** Parameters of the equipment on which the samples were made [83,84].

Manufacturing Method	HP Multi Jet Fusion	Selective Laser Sintering
3D Printer	HP MJF 5200	EOS P396
Building volume (mm)	380 × 284 × 380	340 × 340 × 600
Building speed (m/s)	Up to 0.014	Up to 6
Layer thickness (mm)	0.08	0.12
Working platform temperature (°C)	180–185	168–175
Powder feeder temperature (°C)	N/A (uniform heating)	165–170
Melting point (°C)	178–182	185
Sintering energy source	Heating lamps	Laser CO_2_
Power (W)	Variable (IR array)	30–50
Hatch distance (mm)	N/A (full layer exposure)	0.15–0.25
Beam offset (mm)	N/A (agent-based control)	0.0–0.1
Energy density (J/mm^2^)	Variable (agent-controlled)	0.04–0.08
Average power consumption (kW)	12	2.4
Power supply	380–415 V, 50 A max	400 V/32 A
Pre-processing	No gas required	Nitrogen purging required
3D printing process	Inkjet agents + IR heating	Laser sintering layer-by-layer

**Table 4:** Parameter post-processing machines of the equipment on which the samples were made [85,86,87,88].

Post-Processing Method	Cleaning	Surfacing	Dyeing	Chemical Smoothing
Machine	DyeMansion Powershot C	DyeMansion Powershot S	DyeMansion DM60	DyeMansion Powerfuse S
Print-to-product workflow	Cleaning	PolyShot Surfacing	DeepDye Coloring	VaporFuse Surfacing
Automation	Automated processes and manual loading/unloading	Individual programming, control, and monitoring of process parameters via control panel and touch screen	Automated processes and manual loading/unloading	Automated processes and manual loading
Process chamber volume	26 l | 6.8 gal	26 l | 6.8 gal	80 l | 21.1 gal	85 l | 22.5 gal
Capacity per run	Up to 3/4 EOS P396, 1× HP Jet Fusion 4200/5200 or close to 1.5 full-sized Stratasys H350 build jobs	Up to 3/4 EOS P396, 1× HP Jet Fusion 4200/5200 or close to 1.5× full-sized Stratasys H350 build jobs	Up to 3/4 EOS P396, 1× HP Jet Fusion 4200/5200 or close to 1.5 full-sized Stratasys H350 build jobs	Manual loading belt: 705 mm × 1700 mm × 850 mm | 27.8 inch × 66.9 inch × 33.5 inch
Cycle time	3 to 10 min	Variable from 1 to 40 min, typically 10 to 15 min	150 min	45 to 180 min
Compatible 3D printing technology	SLS, SAF, MJF, HSS	SLS, SAF, MJF, HSS	SLS, SAF, MJF, and HSS	Rigid materials like PA11 or PA12, and semi-rigid materials like TPU from most common printing technologies and material suppliers

**Table 5:** Configuration of manufactured specimens in SLS and HP MJF technologies, together with the post-processing treatment undergone.

Technology	Material	3D Printing Machine	Post-Processing Method	Type of Sample and Their Purpose	Samples
SLS	PA2200	EOS P396	Powershot C	Tensile strength test specimens 1. For reference samples; 2. For ageing 6 months; 3. For ageing 12 months -6 specimens for each test	18
			Powershot C + DM60 + Powershot S		18
			Powershot C + DM60 + Powershot S + Powerfuse S		18
HP MJF	PA12 HP 3D High Reusability	HP 5200	Powershot C		18
			Powershot C + DM60 + Powershot S		18
			Powershot C + DM60 + Powershot S + Powerfuse S		18

**Table 6:** Statistical description of the tensile strength, Young’s modulus, and elongation at break of the SLS and MJF specimens for the DyeMansion Powershot C + Powershot S post-processing treatment during the reference, 6- and 12-month ageing periods.

Technology	Post-Processing	Ageing Test	σ (MPa)			E (GPa)			ε (%)		
			Mean	Std	CV	Mean	Std	CV	Mean	Std	CV
SLS	Powershot C + Powershot S	Reference	45.24	0.25	0.54	1.48	0.02	1.47	12.84%	0.42%	3.61%
		1S—6 months	45.20	0.30	1.49	1.53	0.05	3.49	11.2%	1.2%	10.7%
		2S—12 months	43.50	0.40	0.87	1.48	0.05	3.49	9.51%	0.61%	6.35%
HP MJF	Powershot C	Reference	43.49	1.56	3.60	1.48	0.03	1.99	9.52%	0.56%	5.89%
		1S—6 months	42.30	1.40	3.33	1.50	0.09	6.18	7.9%	0.7%	8.8%
		2S—12 months	28.90	7.10	24.39	1.49	0.08	5.26	3.20%	2.30%	24.40%

**Table 7:** Statistical description of the tensile strength, modulus of elasticity, and elongation in tension of SLS and MJF samples for DyeMansion Powershot C + DM60 + Powershot S finishing during the reference, 6- and 12-month ageing periods.

Technology	Post-Processing	Ageing Test	σ (MPa)			E (GPa)			ε (%)		
			Mean	Std	CV	Mean	Std	CV	Mean	Std	CV
SLS	Powershot C + DM60 + Powershot S	Reference	43.72	1.03	2.36	1.41	0.04	3.48	12.75%	0.60%	5.21%
		1S—6 months	44.80	0.60	1.25	1.40	0.04	2.74	14.8%	0.9%	6.4%
		2S—12 months	45.00	0.60	1.40	1.45	0.03	1.87	12.50%	0.80%	6.51%
HP MJF	Powershot C+DM60+ Powershot S	Reference	44.90	0.98	2.19	1.39	0.03	2.12	12.90%	0.94%	7.27%
		1S—6 months	45.00	0.40	0.81	1.36	0.03	2.02	12.2%	0.8%	6.2%
		2S—12 months	31.40	3.60	11.59	1.66	0.02	1.46	2.50%	0.60%	21.53%

**Table 8:** Statistical description of the tensile strength, modulus of elasticity, and elongation in tension of SLS and MJF samples for DyeMansion Powershot C + DM60 + Powershot S + Powerfuse S finishing at baseline, 6- and 12-month ageing.

Technology	Post-Processing	Ageing Test	σ (MPa)			E (GPa)			ε (%)		
			Mean	Std	CV	Mean	Std	CV	Mean	Std	CV
SLS	Powershot C+DM60+ Powershot S + Powerfuse S	Reference	44.47	0.70	1.57	1.29	0.12	9.02	16.13%	2.56%	15.84%
		1S—6 months	40.40	0.38	0.94	0.89	0.02	2.04	18.2%	1.7%	9.3%
		2S—12 months	35.52	5.98	16.84	1.14	0.12	10.46	8.31%	4.96%	59.70%
HP MJF	Powershot C+DM60+ Powershot S + Powerfuse S	Reference	44.85	0.92	2.04	1.28	0.03	2.52	14.28%	1.94%	13.56%
		1S—6 months	26.10	1.00	3.82	1.17	0.02	1.95	3.2%	0.2%	6.3%
		2S—12 months	20.53	1.64	8.00	1.23	0.04	3.51	2.01%	0.29%	14.70%

**Table 9:** Three-factor ANOVA results for tensile strength (σ) and Young’s modulus (*E*).

Factor	F(σ)	p(σ)	$\eta_{\mathrm{partial}}^{2}\left(\sigma \right)$	F(E)	p(E)	$\eta_{\mathrm{partial}}^{2}\left(E\right)$
Technology	162.9	<0.0001	0.69	101.2	<0.0001	0.58
Post-processing variant	72.3	<0.0001	0.67	192.6	<0.0001	0.89
Ageing time	130.9	<0.0001	0.78	89.1	<0.0001	0.61
Technology × Post-processing variant	21.4	<0.0001	0.43	15.9	<0.0001	0.33
Technology × Ageing time	34.1	<0.0001	0.52	25.4	<0.0001	0.41
Variant × Ageing time	15.2	<0.0001	0.35	14.8	<0.0001	0.32
Technology × Post-processing variant × Ageing time	10.7	<0.0001	0.29	9.2	<0.0001	0.22

**Table 10:** Three-factor ANOVA results for elongation at break (ε).

Factor	F(ε)	p(ε)	$\eta_{\mathrm{partial}}^{2}\left(\varepsilon \right)$
Technology	144.8	<0.0001	0.77
Post-processing variant	85.2	<0.0001	0.70
Ageing time	138.7	<0.0001	0.79
Technology × Post-processing variant	18.3	<0.0001	0.39
Technology × Ageing time	29.1	<0.0001	0.50
Variant × Ageing time	17.0	<0.0001	0.36
Technology × Post-processing variant × Ageing time	11.5	<0.0001	0.27

**Table 11:** Multivariate MANOVA results for mechanical properties (σ, *E*, ε).

Effect	Wilks’ λ	*F*	*p*
Technology	0.41	25.3	<0.0001
Post-processing variant	0.32	29.6	<0.0001
Ageing time	0.275	21.14	<0.0001
Technology × Post-processing variant	0.86	2.1	0.11
Technology × Ageing time	0.51	7.4	<0.0001
Variant × Ageing time	0.42	5.9	<0.0001
Technology × Post-processing variant × Ageing time	0.38	4.8	<0.0001

**Table 12:** Principal component loadings and explained variance for tensile strength (σ), Young’s modulus (*E*), and elongation (ε).

Main Component	Load (*σ*)	Load (*E*)	Load (*ε*)	Explained Variance (%)	Cumulative Variance (%)
PC1	0.78	0.22	0.81	74.1	74.1
PC2	0.10	0.92	0.14	21.3	95.4

**Table 13:** Selected results of post hoc comparisons: statistically significant differences in mechanical properties (with *p*-values and Cohen’s *d* effect sizes).

Comparison	Properties	*p*	Cohen’s *d*
MJF vs. SLS (Powerfuse S, 12 months)	Ultimate tensile strength	<0.0001	3.4
MJF 12 months vs. Ref (Powerfuse S)	Ultimate tensile elongation	<0.0001	5.1
MJF 12 months vs. Ref (DM60 + Powershot S)	Ultimate tensile elongation	<0.0001	4.2
MJF vs. SLS (Powershot C+S, 12 months)	Ultimate tensile strength	<0.0001	2.8

## Data Availability

The original contributions presented in this study are included in the article. Further inquiries can be directed to the corresponding author.

## Abbreviations

The following abbreviations are used in this manuscript:

SLS	Selective Laser Sintering
HP MJF	HP Multi Jet Fusion
AM	Additive Manufacturing
PMMA	polymethyl methacrylate
TPU	thermoplastic polyurethane
FFF	Fused Filament Fabrication
SLA	Stereolithography
PA12	Polyamide 12 (HP 3D High Reusability, HP trademark; PA2200, EOS trademark)
Std	Standard Deviation
CV	Coefficient of Variation
PSC SLS	Manufactured in SLS and post-processed in DyeMansion Powershot C
PSC HP MJF	Manufactured in HP MJF and post-processed in DyeMansion Powershot C
PCS_DM_HP_MJF	Manufactured in HP MJF and post-processed in DyeMansion Powershot C + DM60 + Powershot S
PCS_DM_SLS	Manufactured in SLS and post-processed in DyeMansion Powershot C + DM60 + Powershot S
PWS_HP_MJF	Manufactured in HP MJF and post-processed in DyeMansion Powershot C + DM60 + Powershot S + Powerfuse S
PWS_SLS	Manufactured in SLS and post-processed in DyeMansion Powershot C + DM60 + Powershot S + Powerfuse S
MDR	Medical Device Regulation
EU	European Union
σ	Ultimate tensile strength
*E*	Young modulus
ε	Tensile elongation at break
*F*	A measure of effect strength in ANOVA; higher values indicate larger differences between groups relative to within-group variability
*p*	The significance level; shows the probability that the observed differences occurred by chance (p<0.05 indicates statistical significance)
Partial $\eta^{2}$	A measure of effect size; indicates the proportion of variance in the dependent variable explained by a given factor
Cohen’s *d*	A standardized effect size; quantifies the difference between two group means in terms of pooled standard deviation (larger values mean stronger effects)
